# Multitemporal Observations of Sugarcane by TerraSAR-X Images

**DOI:** 10.3390/s101008899

**Published:** 2010-09-28

**Authors:** Nicolas Baghdadi, Rémi Cresson, Pierre Todoroff, Soizic Moinet

**Affiliations:** 1 CEMAGREF, UMR TETIS, 500 rue François Breton, 34093 Montpellier cedex 5, France; E-Mails: remi.cresson@teledetection.fr (R.C.); soizic.moinet@teledetection.fr (S.M.); 2 CIRAD-Reunion, Ligne Paradis, 97410 Saint-Pierre, France; E-Mail: pierre.todoroff@cirad.fr (P.T.)

**Keywords:** SAR, TerraSAR-X, sugarcane, NDVI, Reunion Island

## Abstract

The objective of this study is to investigate the potential of TerraSAR-X (X-band) in monitoring sugarcane growth on Reunion Island (located in the Indian Ocean). Multi-temporal TerraSAR data acquired at various incidence angles (17°, 31°, 37°, 47°, 58°) and polarizations (HH, HV, VV) were analyzed in order to study the behaviour of SAR (synthetic aperture radar) signal as a function of sugarcane height and NDVI (Normalized Difference Vegetation Index). The potential of TerraSAR for mapping the sugarcane harvest was also studied. Radar signal increased quickly with crop height until a threshold height, which depended on polarization and incidence angle. Beyond this threshold, the signal increased only slightly, remained constant, or even decreased. The threshold height is slightly higher with cross polarization and higher incidence angles (47° in comparison with 17° and 31°). Results also showed that the co-polarizations channels (HH and VV) were well correlated. High correlation between SAR signal and NDVI calculated from SPOT-4/5 images was observed. TerraSAR data showed that after strong rains the soil contribution to the backscattering of sugarcane fields can be important for canes with heights of terminal visible dewlap (htvd) less than 50 cm (total cane heights around 155 cm). This increase in radar signal after strong rains could involve an ambiguity between young and mature canes. Indeed, the radar signal on TerraSAR images acquired in wet soil conditions could be of the same order for fields recently harvested and mature sugarcane fields, making difficult the detection of cuts. Finally, TerraSAR data at high spatial resolution were shown to be useful for monitoring sugarcane harvest when the fields are of small size or when the cut is spread out in time. The comparison between incidence angles of 17°, 37° and 58° shows that 37° is more suitable to monitor the sugarcane harvest. The cut is easily detectable on TerraSAR images for data acquired less than two or three months after the cut. The radar signal decreases about 5dB for images acquired some days after the cut and 3 dB for data acquired two month after the cut (VV-37°). The difference in radar signal becomes negligible (<1 dB) between harvested fields and mature canes for sugarcane harvested since three months or more.

## 1. Introduction

Sugarcane is one of the most important crops in the tropics, with a global production estimated at 1,250 million tons a year and a cropped area of about 20 millions hectares [1]. This crop is usually cultivated for five to seven years before a new planting. On Reunion Island (located in the Indian Ocean) a first cutting (portion of stem) is planted and grows according to five growth stages that constitute a cycle of 12 to 14 months. These stages are 1—the germination phase with development of roots and primary stems, 2—the tillering phase, where secondary stems develop from underground buds and later become the first visible stems from the surface (they form a group of stems but are born from a single cutting). The next phases include the grand growth phase, 4—the Maturation and ripening phase and sometimes 5—the flowering phase with the transformation of the terminal bud into a floral bud. At maturation the cane height reaches 4–5 m according to cane cultivated variety. After the harvest, new stems grow from the stubble buds and a second cycle begins until the next harvest. Because the yield decreases with successive cycles, the same sugarcane plantation is generally harvested five to seven times.

Sugarcane was introduced on Reunion Island at the 17th century. At present, it is the most important crop of the island. Indeed, it represents 60% of the useful agriculture area, what corresponds to approximately 26,000 hectares which are distributed on many small farms mainly located on the circumference of the Island, at a maximum altitude of 800 m. The light and water requirements of the cane necessitate planting at the end of the year and a harvest between July and December. Indeed, on Reunion Island, there are two main seasons: the hot rainy season from November to April, and the cool dry season from May to October.

One of the main needs expressed by sugarcane industries is to have information on the harvest progress throughout the harvest season [2,3]. The dynamic mapping of sugarcane harvest on a large spatial scale allows optimized cutter deployment, transport operations, efficiency of factories, and finally permits a better estimation of the effective yield.

Several studies demonstrated the potential of optical multi-temporal images for monitoring sugarcane harvest [4–7]. However, the use of optical images is sometimes limited because of atmospheric conditions (clouds). Indeed, the interval between two cloud-free images is sometimes too long (more than 2 months); this makes difficult the discrimination between a standing crop and the regrowth in a field harvested at the beginning of the harvest campaign. In active microwave remote sensing, Synthetic Aperture Radar (SAR) provides measurements day and night, regardless of meteorological conditions. With their frequent revisits, SAR sensors are very useful remote sensing data sources for agriculture monitoring in tropical regions. Moreover, the radar signal is very sensitive to soil and vegetation parameters [8,9]. Short SAR wavelengths such as X-band (∼3 cm) and C-band (∼6 cm) interact mainly with the top part of the canopy layers while long wavelengths such as L-band (∼20 cm) and P-band (∼100 cm) have a high penetration depth and can thus penetrate into the vegetation cover and reach the soil [8]. This penetration depth depends on vegetation parameters (e.g., water content, leaf size, stem density, LAI). The new generation SAR sensors such as TerraSAR-X allow the acquisition of images at very high spatial resolution (∼1 m). Moreover, it has a high revisit interval making it possible to monitor the harvest with high temporal frequency (daily to weekly).

For several years, many studies have shown the usefulness of Synthetic Aperture Radar data for crop parameter retrieval [10–16], but very few were applied to sugarcane [17,18]. Most of these studies were carried out using C-band SAR due to the availability of this radar frequency on the first generation of satellite SAR sensors (ERS-1/2, RADARSAT-1, ASAR/ENVISAT). From several theoretical analyses and experimental observations carried out on vegetated soil, the backscattering coefficient at HV polarization was showed suitable for monitoring the growth of vegetation [19,20]. The comparison between the backscattering coefficient at C-, L-, and P-bands for estimating leaf area index of some types of crops (alfalfa, wheat, corn) showed that the highest informational content is included in the L-band [21]. Moreover, shallow angle data (>40°) was better correlated to rice crop height than steep angle (20°) data using RADARSAT-1 (C-band) [13,16].

The analysis by Inoue *et al*. [14] of microwave backscattering coefficients in five frequencies (Ka, Ku, X, C, and L) over paddy rice field showed that LAI was best correlated with the C-band (HH and cross polarizations) and biomass was best correlated with the L-band (HH and cross polarizations). Contrarily, shorter wavelengths (Ka, Ku, and X bands) were poorly correlated with LAI and biomass. Bouman [11] found the possibility of crop parameter estimation from X-band radar backscattering measurements was very low. This low potential is attributed to the early saturation of the radar signal with crop parameters. Radar measurements of rice crop using X-band [22] showed that the backscattering coefficient reached its peak value (for plant height around 60 cm) before the maximum height of the plant is reached (∼100 cm). Recently, Lin *et al*. [18] proposed a method to map sugarcane growth and estimate sugarcane leaf area index (LAI) by using the ratio of ASAR HV to HH data (C-band). The results showed that C-band SAR data are very promising for monitoring sugarcane growth. A first study on the potential of TerraSAR data for monitoring sugarcane crops on Reunion Island was carried out by Baghdadi *et al*. [17]. Harvested fields were easily detected on ASAR images if the SAR acquisition was close to harvest date. The limited database in X-band (only two images) did not allow for analysis of the potential of X-band for monitoring sugarcane crop growth and for mapping harvested fields. From the TerraSAR images studied, results showed good correlation between radar signal and cane heights between 0 and approximately 100 cm at 53° (HH polarization).

This study examined the relationship between TerraSAR signal and sugarcane height as a function of instrumental parameters (polarization and incidence), and precipitation. In addition, the potential of TerraSAR-X for mapping harvested sugarcane crop was studied. The correlation between radar signal and Normalized Difference Vegetation Index (NDVI) extracted from SPOT images was also analyzed.

## 2. Study Area and Data Used

### 2.1. Study Area

**Figure 1.**
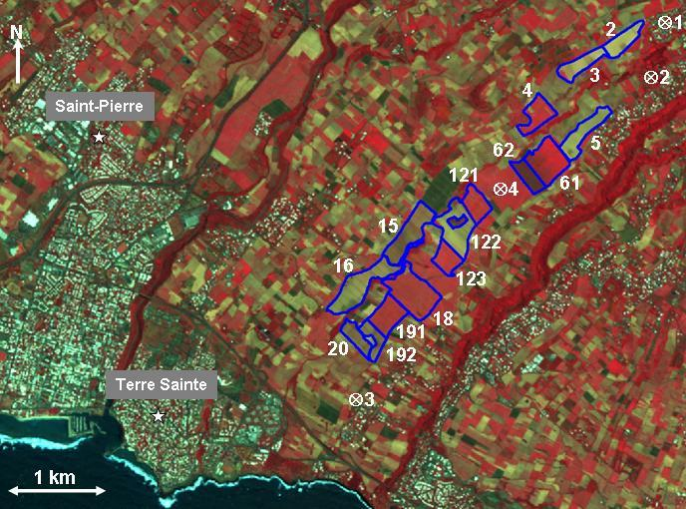
A false color composite of a SPOT-5 image acquired over the study site in Reunion Island on October 21, 2008 (Red: band-3; Green: band-2; Blue: band-1). Reference sugarcane fields are outlined in blue. “⊗” indicates the location of meteorological stations (⊗1: Bérive-2; ⊗2: Isautier-Bérive; 3⊗: Isautier-Foyer; ⊗4: Isautier-Ringuin).

The study site covers a sugarcane farm located in the south of Reunion Island, close the town of Saint Pierre (latitude: 21°19′ S - longitude: 55°31′ E; Figure 1). The study site is composed mainly of agricultural fields intended for growing sugarcane with soil composed of 50% clay, 30% silt, and 20% sand [23]. Fifteen sugarcane fields of an average size of 9 ha were studied: {2, 3, 4, 5, 61, 62, 121, 122, 123, 15, 16, 18, 191, 192, 20}. These training fields extend on 4.5 km approximately, between 100 m to 500 m altitude. TerraSAR-X and SPOT images were acquired over our study site. The images belong to the KALIDEOS database set up by the CNES (French Space Agency) [24,25].

### 2.2. TerraSAR Date

Sixty-four TerraSAR-X images (X-band ∼ 9.65 GHz) were acquired between the 14th of December 2008 and the 20th of January 2010 with different incidence angles (17°, 31°, 37°, 47° and 59°), and in mono- and dual-polarization modes (HH, VV, HH/VV, HH/HV, VH/VV). The imaging modes used were Spotlight and Stripmap. The pixel spacing of TerraSAR images was between 1 and 3 m. Characteristics of TerraSAR images used in this study are summarized in Table 1.

Radiometric calibration using MGD (Multi Look Ground Range Detected) TerraSAR images was carried out using the following equation [26]:

(1) $$\sigma_{i}^{{}^{\circ}}(\mathrm{dB})=10{\text{log}}_{10}(\mathrm{Ks}.{\mathrm{DN}}^{2}-\mathrm{NEBN})+10{\text{log}}_{10}(\text{sin}\theta_{i})$$

This equation transforms the amplitude of backscattered signal for each pixel (*DN_i_*) into a backscattering coefficient (σ°*_i_*) in decibels. The calibration coefficient *Ks* (scaling gain value) varies within the range of 7.7 × 10^−6^ to 1.2 × 10^−5^, depending on radar incidence angle (θ_i_) and polarization (low values for cross-polarizations or high incidences). It is given in the section “calibration” of the TerraSAR data delivery package. NEBN is the Noise Equivalent Beta Naught. It represents the influence of different noise contributions to the SAR signal. The NEBN is described using a polynomial scaled with *Ks*. The polynomial coefficients are derived from the TerraSAR product file (section “noise” of SAR data delivery package). The absolute radiometric accuracy of TerraSAR data is 0.6 dB [15]. All TerraSAR images were then georeferenced using GPS points (cubic convolution resampling algorithm). The RMS georeferencing accuracy varies from 1.2 to 1.6 pixels.

The *NEBN* varies from −26.8 to −22.3 dB for HH-17° and VV-17° in mono- and dual-polarization modes. For images at 31°, the *NEBN* varies from −26.4 to −23.9 dB for HH and VV polarizations in mono-polarization mode, and from −19.1 to −12.2 dB for HH, HV, and VV polarizations in dual-polarization mode. For 59°, the NEBN varies from −21.9 to −21.4 dB for HH and VV polarizations in mono-polarization mode, and from −20.0 to −13.3 dB for HH, HV, and VV polarizations in dual-polarization mode. In Spotlight mode, the *NEBN* varies between −26.8 to −18.6 dB whereas in Stripmap mode, it varies between −19.1 to −12.2 dB.

The high values of NEBN found for images acquired in Stripmap mode did not allow a calibration of many pixels because the term Ks.DN^2^ was lower than the noise NEBN. This problem is very frequent for pixels corresponding to smooth areas (specular reflection), such as harvested fields. Moreover, the results show that the influence of the noise is stronger for cross-polarizations than for co-polarizations because even if the NEBN is of the same order of magnitude for cross- and co-polarizations, the term Ks.DN^2^ is lower for cross-polarizations. Many pixels impossible to calibrate because Ks.DN^2^ < NEBN were also observed at high incidence angles (until 30% of image pixels). These pixels were not used in the calculation of the statistics, what represents a strong loss of information.

Speckle noise, due to the coherent interference of waves reflected from many elementary scatterers, is present on SAR images and makes the pixel-by-pixel interpretation of SAR images extremely difficult. This explains why the analysis of radar signals is generally carried out on homogeneous areas with several pixels or at field scale (which helps reduce speckle). In practice, the mean backscattering coefficients were calculated from calibrated SAR images by averaging the linear σ° values of all pixels within reference fields or (sub-fields in the case where only a part of field is harvested).

### 2.3. SPOT Data

Fifteen SPOT-4/5 images were acquired over Reunion Island between July 24, 2008 and December 22, 2009 with spatial resolution of 20 and 10 m (Table 2). All images were orthorectified and coregistered to the UTM coordinate system (zone 40 South) with a root mean square error of less than 0.5 pixel. The radiometry of the images was atmospherically corrected using the 6S code (Kalideos), so that pixel values represented top of canopy reflectances (TOC) in the four spectral bands. The Normalized Difference Vegetation Index (NDVI) was calculated from optical images for each reference field to determine the state of the fields at the acquisition time of the SPOT images (fields in vegetation or harvested).

**Figure 2.**
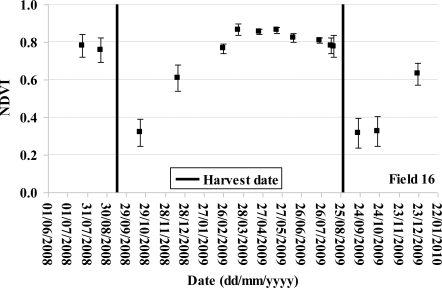
Example of the temporal profile of sugarcane NDVI. Field 16 was harvested on September 01, 2008 and August 29, 2009. The error bars correspond to the standard deviation of NDVI.

The NDVI temporal profile of sugarcane fields can be divided into two periods: a period in which NDVI values increase, corresponding to the vegetative development of the sugarcane, and another period with steady or decreasing values, corresponding to the mature phase of the plant. Figure 2 shows an example of the temporal profile of NDVI for the reference sugarcane field 16. The low value of NDVI on October 21, 2008 and September 21, 2009 is correlated to harvesting dates (September 01, 2008 and August 29, 2009). After these dates, the NDVI increases illustrating the growth of the cane.

### 2.4. Ground Measurements

Ground truth measurements of sugarcane height were performed on several reference fields from November 07, 2008 to June 06, 2009. On each reference field, two experimental areas of 1.5 m × 1.5 m were used to collect the sugarcane height (mean cane height of all cane plants inside of experimental areas), number of stems and leaves. Ground measurements showed that the sugarcane in our study site grows about 25 cm per month during the five first months, 40cm between the 6th and 9th month, and then of about 10–20 cm per month until reaching the mature height of the cane. The ground measurements of the sugarcane height correspond to the height of terminal visible dewlap (htvd). They exclude the leafy tops which have heights of the order of 55 cm for sugarcane with htvd of 20 cm, 105 cm for htvd of 50 cm, and of 125 cm for htvd between 100 and 180 cm. Beyond htvd of 180 cm, the leafy top height is about 135 cm. For our reference fields, the mean number of stems and leaves was about 17 and 77 per m^2^, respectively (with a standard deviation of about 7 and 30, respectively). Some biophysical parameters of sugarcane are shown in Table 3.

In addition, the farmer of our reference fields also provided the harvesting dates of each reference field. Daily precipitation data recorded at four meteorological stations located on the farm were also used: Bérive-2, Isautier-Bérive, Isautier-Foyer, and Isautier-Ringuin. The effect of soil moisture content was taken into account in this study using precipitation data. Indeed, soil moisture measurements were difficult to carry out because the terrain is inaccessible in rainy weather and the soil is covered with mulch (dead leaves).

## 3. Results and Discussion

### 3.1. Sensitivity of Radar Signal to Sugarcane Height

**Figure 3.**
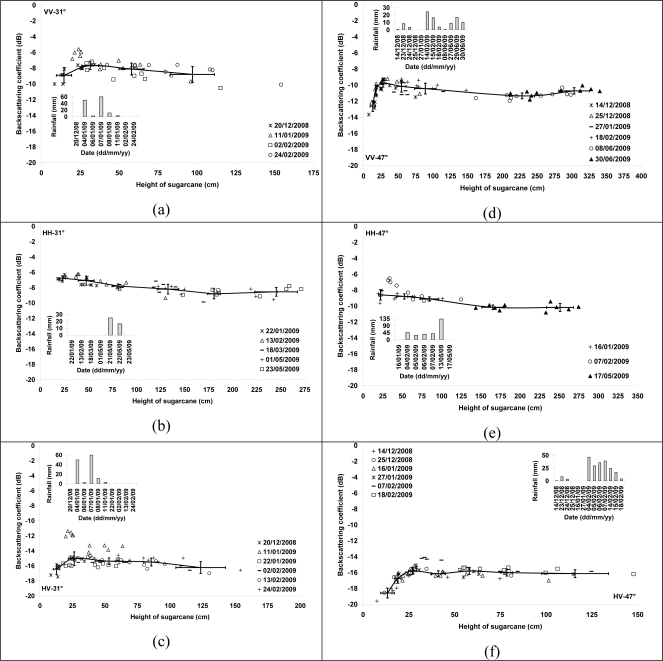
Radar backscattering coefficient as a function of plant height for VV, HH, and HV polarizations and incidence angle of 31° and 47°. The sugarcane height corresponds to the height of terminal visible dewlap (htvd). Bars represent standard deviation of the mean.

The sensitivity of TerraSAR-X signals has been analyzed as a function of sugarcane height (htvd). Results show that the radar signal increases with the sugarcane height for the fields at the beginning of growth (htvd and total cane height respectively lower than 50 cm and 155 cm, depending on incidence angle and polarization) (Figure 3). The growth of the sugarcane leads to an increase in height, number and size of leafs, and number and size of stems. This involves an increase of volume backscattering coefficient as well as attenuation of radar signal. However, the increase and decrease of backscatter caused by volume scattering and attenuation at the same time make radar signal reach saturation and then decrease when plant height is larger than 50 cm. The dynamic of radar signal with the sugarcane height is slightly higher at 47° than at 31°. A dynamic of 5 dB for 47° and 2.5 dB for 31° is observed for cane heights between 0 and approximately 50 cm. Results show a clear increase in the radar signal after rainy episodes, in particular for young canes. Descriptive error bars are used to summarize the distribution of our data. Statistical analysis of the radar signal is carried out in using different ranges of sugarcane height. The standard deviations (SD) error bars show that the backscattering coefficient data are spread at about ±1 dB (Figure 3).

#### 3.1.1. TerraSAR data at 31°

With an incidence angle of 31°, the radar signal increases 2.5 dB with sugarcane height for cane lower than 30 cm (1–2 months old). This increase did not depend on the polarization (VV and HV). After this threshold, a decrease of radar signal is observed (Figures 3a, 3b, 3c).

The increase in σ° for the image on January 11, 2009 for the fields with cane heights lower than 30 cm (2 dB in VV and 3 dB in HV) is correlated with the strong precipitation recorded between January 4, and January 11, 2009 (130 mm) (Figures 3a, 3c). This increase is probably due to the increase of the soil contribution (influenced by soil moisture) in the total radar signal. The smaller radar incidence angle (31°) and the low vegetation cover (young canes) allowed this strong contribution from the soil.

Beyond 40 cm, results show that the radar signal in VV polarization of the same order of magnitude as the other dates, which is explained by a weak contribution of the soil due to a low penetration of the radar wave in the vegetation cover. In HV polarization, the radar signal on the image of January 11, 2009 remains higher of approximately 1.5 dB than the other dates for cane heights between 40 and 65 cm. For canes of approximately 100cm height, the soil contribution to the radar signal becomes very weak.

The radar image of May 23, 2009 (HH-31°) confirms that the contribution of the soil becomes negligible for mature canes (heights between 150 and 230 cm). Indeed, the radar signal on May 23, 2009 is of the same order of magnitude as that of other dates in spite of 42mm of precipitations two days before the radar acquisition.

Cookmartin *et al.* [27] showed for wheat fields that the early season backscattering coefficient values are dominated at C-band by direct soil backscatter (to the 111th day after germination). From day 111, the plant stems attenuate the soil term. The radar signal is then dominated by the scattering properties of the leaves and stems at day 146 (after germination) and by the ears alone from day 181. Schoups *et al*. [28] studied the sensitivity of soil and crop parameters on the radar backscattering at C-band (5.3 GHz) of sugar beet fields in using Lang’s and Karam’s microwave scattering models [29,30]. Results showed that for sugar beet with 15 cm height, both ground and canopy parameters are important. The influence of the soil became small for a fully grown sugar beet canopy (height = 50 cm).

#### 3.1.2. TerraSAR data at 47°

The radar signal increases with the sugarcane height until approximately 50 cm (2–3 months of age) and then decrease. This increase in the signal about 5 dB did not seem to depend on the polarization (Figures 3d, 3e, 3f). The strong rains which preceded TerraSAR acquisitions of February 18, 2009 (45 mm in 5 days) and June 30, 2009 (38 mm in 3 days) did not involve a notable increase in the radar signal for these two images in comparison to other images dates. The cane height varied from 50 to 150 cm on February 18, 2009 and from 210 to 340 cm on June 30, 2009. A cane height of 50 cm combined with a high radar incidence (47°) seems to be the main reason to the weak penetration of the radar signal in the vegetation cover, and consequently to the weak soil contribution to total radar signal.

The TerraSAR image of February 07, 2009 (HH and HV) shows that the radar signal increases after strong rains about 1.5 dB for canes of 30–40 cm. Beyond 40 cm, the soil contribution decreases with the height and the radar signal radar becomes independent of soil characteristics. Indeed, at the radar acquisition date of February 07, 2009, the soil was very wet with the accumulated rains of approximately 150 mm over 4 days. The image of May 17, 2009 was also acquired under very wet conditions (133 mm of rain the May 13, 2009) whereas the analysis of radar signal showed a negligible soil contribution because the cane height varied from 145 to 275 cm. Moreover, the soil contribution seems to be of the same order of magnitude for all polarizations.

#### 3.1.3. TerraSAR data at 17° and 58°

**Figure 4.**
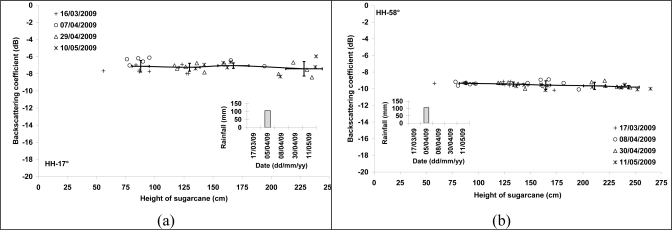
Radar backscattering coefficient as a function of plant height for HH polarization and incidence angles of 17° and 58°. The sugarcane height corresponds to the height of terminal visible dewlap (htvd). Bars represent standard deviation of the mean.

It was difficult to analyze deeply the sensitivity of TerraSAR-X data at 17° and 58° because our database did not contain SAR images acquired for low sugarcane heights (less than 50 cm). For sugarcane heights between 50 and 240 cm, the radar backscattering coefficient remains constant (Figure 4). The strong rain on April 05, 2009 (105 mm) involved a light increase of radar signal on the SAR image of April 07 (17°) for canes of 75 to 95 cm height (∼1 dB). For higher heights (>160 cm), the radar signal remained of the same order as the other SAR dates (Figure 4a). On the image of April 08, 2009, the rain of April 05 did not affect the radar signal for cane heights higher than 75 cm because of the weak contribution of soil for high incidence angle (58°).

#### 3.1.4. Ratios of polarizations and incidences

**Figure 5.**
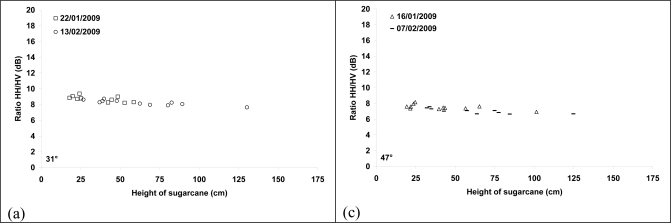

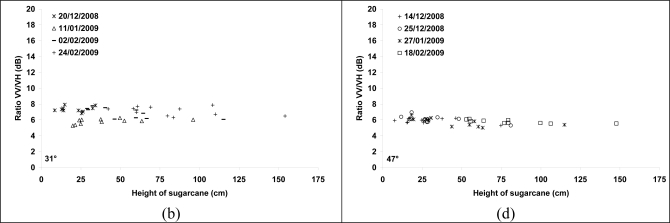
Ratio of TerraSAR-X signals HH/HV and VV/VH and at 31° and 47°. The sugarcane height corresponds to the height of terminal visible dewlap (htvd).

Figure 5 shows that the ratios HH/HV and VV/VH at 31° and 47° decreases slightly with the sugarcane height of about 1 dB for sugarcane height between 10 and 150 cm. VV/VH ratio at 31° is correlated to precipitation because it is lower from 1 to 2 dB (according to cane height) on the image of January 11, 2009 in comparison to other image dates. Indeed, the soil is probably very wet on January 11, 2009 with strong rains one week before the TerraSAR acquisition (130 mm) (Figure 5b). For an incidence of 47°, HH/HV and VV/VH ratios did not seem to depend of precipitation even for low cane heights (10 cm). Indeed, the TerraSAR signal at 47° is of the same order for all TerraSAR dates, even if the images of February 07 and 18, 2009 were acquired under wet conditions (150 mm and 45 mm of accumulated rains over 4–5 days before the SAR acquisitions of February 07 and 18, respectively) while the other images were acquired without rains and thus probably lower soil moisture (December 14 and 25, 2008, January 16 and 27, 2009).

**Figure 6.**
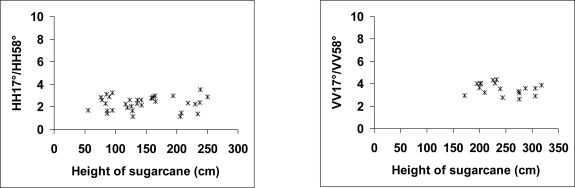
Ratio of TerraSAR-X signals HH17°/HH58° and VV17°/VV58°. The sugarcane height corresponds to the height of terminal visible dewlap (htvd).

Moreover, the ratios HH17°/HH58° and VV17°/VV58° did not show dependence with the cane height for height values between 55 and 320 cm (Figure 6).

### 3.2. Correlation Between TerraSAR Signal and NDVI

**Figure 7.**
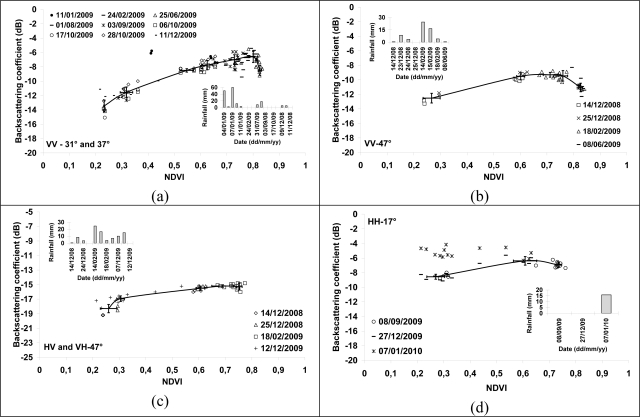
Sensitivity of backscattering coefficient to NDVI. (a) VV-31° and 37°, (b) VV-47°, (c) HV-47°, (d) HH-17°. Bars represent standard deviation of the mean.

The behavior of the TerraSAR signal as a function of NDVI was analyzed (Figure 7). For each TerraSAR image, the backscattering coefficient (σ°) of reference fields was compared to NDVI index calculated from the SPOT image acquired on the date nearest to TerraSAR acquisitions. Only TerraSAR data acquired at ±15 days of SPOT images were analyzed, except for the TerraSAR image of January 11, 2009 where the SPOT image nearest is acquired 24 days before (December 1, 2008). For the comparison between TerraSAR image of January 11 and SPOT image of December 17, the NDVI of SPOT image were increased of 0.1 for NDVI values between 0.3 and 0.7 which corresponds to the increase of NDVI over one month (*cf*. Figure 2). The backscattering coefficient increases with the NDVI index for NDVI smaller than 0.7, followed by a saturation of σ° for values higher than 0.7 (Figure 7). The radar signal increases with the NDVI more quickly for 31° and 37° than for 47° (Figures 7a, 7b, 7c). No correlation is observed between σ° and NDVI for 17° (Figure 7d). The incidence of 31°–37° seems be more correlated to NDVI than 17° and 47°. Results show that the TerraSAR signal at 31°–37° increased by about 7 dB when the NDVI increased from 0.2 (young and harvested sugarcanes) to 0.7 (mature sugarcanes). As VV and VH have a similar sensitivity slope, the ratio VV/VH is independent of NDVI.

Following strong rains a few days before the radar acquisition of January 11, 2009 (accumulated rain of 130 mm in one week), the radar signal increases approximately of 4 dB for the young canes (NDVI = 0.4) and did not change for mature canes (NDVI = 0,65), in comparison to dates far from rainy episodes. An increase of radar signal of about 2 dB on December 11, 2009 (VV-37°) was also observed for the reference fields of low NDVI. This increase is due probably to an increase in soil moisture (high soil contribution) caused by the rain of the two days previous to the radar acquisition (5 mm on December 09 and 6 mm on December 10) (Figure 7a). The strong rain of August 01, 2009 had not modified the radar signal of TerraSAR image (Figure 7a) because the cane was mature and then the soil contribution negligible. In conclusion, the high dynamic of radar signal according to NDVI can be strongly reduced following rainy episodes (increase in the signal for fields with low NDVI values stabilization of signal for fields with high NDVI) for SAR data in X-band, VV polarization, and with incidences between 31° and 37°. This reduction in the signal dynamic can reach 4 dB in the case of strong rains, involving sometimes a strong ambiguity on the cane age because the radar signal corresponding to young cane would be of the same order or higher than that of mature cane.

For an incidence of 47°, the increase in the signal according to NDVI is reduced, with a radar signal dynamic from 3 to 4 dB (according to polarization), for NDVI between 0.25 and 0.7 (Figures 7b, 7c). Results also showed that for 17° of incidence, the radar signal increases with the NDVI of approximately 2.5 dB for NDVI between 0.25 and 0.65 (Figure 7d). For NDVI higher than 0.65, the signal decreases slightly of approximately 1 dB for NDVI between 0.65 and 0.75. The rain of the January 07, 2010 (16 mm) involves a strong increase in the signal of approximately 4 dB for NDVI between 0.2 and 0.3 (−5 dB instead of −9 dB). This increase reached 1dB for NDVI of about 0.55. Thus, following rainy episodes (high soil moisture), the discrimination between young canes (low NDVI) and mature canes (high NDVI) becomes impossible. A slight decrease of NDVI was observed between June and October. For this period, the radar signal remains constant or decreases slightly (Figures 7a, 7b).

### 3.3. Temporal Backscatter and Sugarcane Harvest Detection

The important time series of TerraSAR images at 31° and 37° (20 images over a period of 13 months) makes possible the analysis of temporal behavior of radar signal on a complete cropping cycle. As the radar signal decreases with incidence angle (θ), it was thus necessary to quantify the angular dependence of TerraSAR signal at HH and VV polarizations for incidence angles between 31° and 37°.

In linear units, the radar backscattering coefficient σ° is related to the incidence angle θ by the equation σ° = α*cos*^β^θ [17,31–34]. For a same target (sugarcane field) acquired at two different incidences (θ_1_ and θ_2_), the difference in the radar signal is Δσ° (dB) = 10. β.log_10_(*cosθ_2_*/*cosθ_1_*). First, the parameter β was computed for each reference field using all couples of images acquired at one day of interval except those with precipitation the same day of SAR acquisition. Next, the mean value was calculated for each polarization. Results show β-values of 1.2 for HH and 1.45 for VV (standard deviation of 0.43 and 0.33, respectively). The effect of incidence is about 0.37 dB and 0.45 dB for incidences between 31° and 37° at HH and VV, respectively.

**Figure 8.**
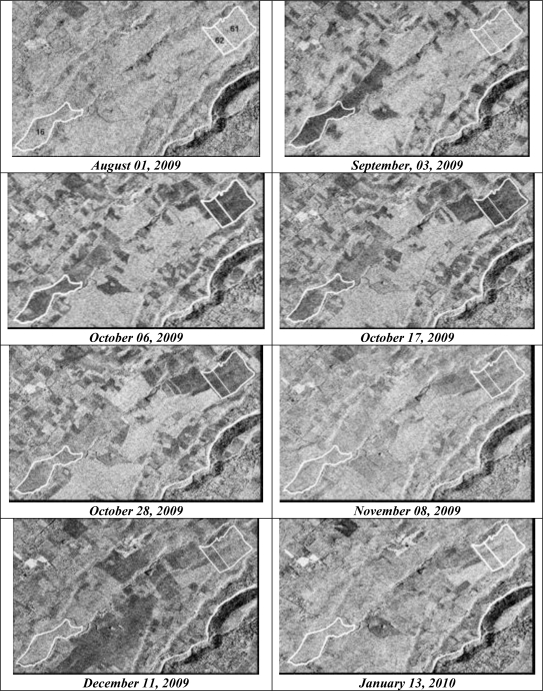
Comparison of several TerraSAR image segments for reference sugarcane fields (61, 62, and 16). All images were acquired at incidence of 37° and in VV polarization. Fields 61, 62, and 16 were harvested on September 10, October 03, and August 29, 2009, respectively.

Figure 8 shows segments of TerraSAR-X images acquired between August 01, 2009 and January 13, 2010 in VV polarization and with an incidence angle of 37°. The interpretation of TerraSAR images shows that the difference between the backscatter of mature cane and of harvested cane is well pronounced at medium incidence angles (37°). The images show high σ° for mature canes and low σ° for harvested fields. The discrimination between harvested fields or young canes (less than two months old) and canes of more than two months old is better with TerraSAR at 31°–37°.

**Figure 9.**
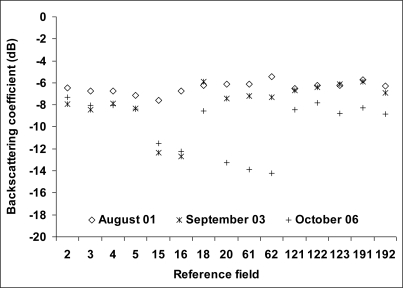
Backscattering coefficient extracted from TerraSAR images of August 01, September 03, and October 06, 2009 (VV-37°). The standard deviation of backscattering coefficients within training fields varies between 1.5 and 2 dB.

Figure 9 shows the temporal variation of σ° for three dates (VV-37°): August 01, September 03, and October 06, 2009. On the image of August 01, the mean radar signal of each reference field was of the same order (∼–7dB) because all fields were mature sugarcanes. On September 03, only fields 15 and 16 had their radar signal decreased (of 5 dB) because these fields were cut the second half of August (August 15 and 29, respectively). One month later, on the image of October 06, the radar signals of fields 15 and 16 were slightly increased (about 1 dB) while those of fields 20, 61 and 62 had strongly decreased (6–7 dB) since they were cut on September 04, September 10, and October 03. Results also showed that σ° of September and October images were in general weaker than in August for mature sugarcanes, what could be due to a drying of the cane. The weakest radar signals (<−11 dB) correspond to harvested sugarcane fields (15, 16, 20, 61, 62) while the strongest values (>−9 dB) correspond to mature canes or to fields harvested more than two months earlier. In conclusion, the low values of σ° correspond to harvested fields and a difference at least of 5 dB was observed between harvested fields and mature fields.

**Figure 10.**
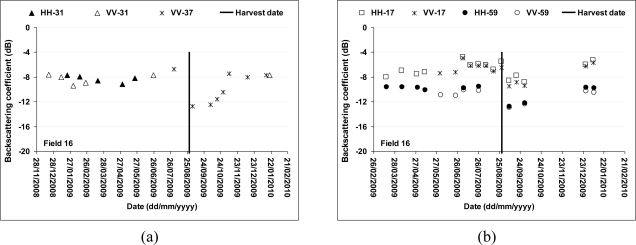
Temporal variation of TerraSAR signal for the reference sugarcane field 16. (a) 31 and 37°, (b) 17° and 59°. Field 16 was harvested on August 29, 2009.

The temporal variation of radar signal was studied for each reference field in VV polarization and with incidences of 31° and 37° An example of the temporal variation of backscatter is given in Figure 10a for reference field 16. The strong decrease in the signal on September 03 is related to the cut of this field approximately one week earlier. Considering one complete cycle of the cane growth, it is possible to observe, from the curve of the backscattering coefficient versus SAR acquisition date (crop age), the σ° variation of a given field between two TerraSAR-X acquisitions. σ° varies from −7 dB on image acquired just before the cut (August 01, 2009) to −13 dB on image of September 03, 2009 which is acquired close the cut of August 29. The potential of TerraSAR images for the monitoring of sugarcane harvesting is demonstrated for data acquired between December 2008 and January 2010 where an important change is observed in the radar signature of Field 16 (Figure 10a). Indeed, this field was cut on August 29, 2009. A decrease in the signal of about 5 dB was observed for this field between its previous state of mature cane and its new state of cut cane. The σ° had decreased of about 5 dB at VV-37° between August 01 and September 03, 2009. This decrease is followed by a weak increase between September 03 and October 28, which corresponds to cane growth with an average cane height of about 40 cm. On the image of October 28 (cut two months earlier), the radar signal was of the order of −10 dB comparatively to a mean level of signal for mature canes of about −8 dB. This difference of 2 dB is the limit from which the cut would be not easily detectable. The high value of signal observed on November 08 (−7 dB instead of about −9.5 dB) is in relation to an excessively rainy week with 111mm of precipitation between the 06th and 08th of November 2009. This result shows the influence of the rain and probably of the soil moisture on TerraSAR signal with an increase of about 2.5 dB for sugarcane of two months and half of old (∼50 cm of height).

Results obtained with incidence angles of 17° and 58° show a weak dynamic of σ° for the mapping of the harvested fields (Figure 10b). Indeed, the maximum difference of the signal between the cut fields and the fields in vegetation is about 3dB. Considering the precedents results, this difference will decrease for an image acquired after a rainy episode. The mapping of cuts would be then very difficult, with confusion between the signals of the two types of surface states: cut and mature cane. Moreover, strong fluctuations of radar signal were observed at low incidence angle (17°) for young canes due to the important contribution of soil to total backscattering.

Baghdadi *et al*. [17] observed a better potential with TerraSAR images at 53° than at 39°. Indeed, in Baghdadi *et al*. [17] the term NEBN had not been used in the calibration process of TerraSAR images. All pixels of very low radar signal (pixels of cut) and which could correspond to NEBN > (KsDN^2^) had not been excluded in the calculation of mean backscattering coefficients within reference fields. So, in Baghdadi *et al*. [17] the mean signal on a cut field was weaker and the difference between mature cane and harvested field higher.

Figure 10b also shows that HH and VV polarizations are strongly correlated. The general trend is that the HH response is slightly higher than the VV (on the order of 1 dB). This confirms the effect of higher attenuation at the VV polarization for sugarcanes with a vertical structure [35].

The increase of the volume contribution as a function of sugarcane growth stage, combined to the decrease of the direct soil contribution, will result in a relative stabilization of the radar signal [18,35]. At the end of the ripening phase a slight decrease of the backscattering coefficient could be observed, corresponding to the plant drying before harvest [36].

To understand the radar backscattering behaviour of sugarcane fields, simulations using MIMICS backscattering model at C-band was analyzed by Lin *et al*. [18]. Simulations results show that the direct backscattering from the soil contributes significantly to the total backscattering in the early growing stage of crop. Indeed, the soil contribution to the total backscattering is of the same order than the direct backscattering from the vegetation layer to growing day 120 (stem height about 130 cm). For mature sugarcanes, the radar backscattering values result mainly from volume scattering. The interactions soil-vegetation, vegetation-soil, and soil-vegetation-soil are negligible.

## 4. Summary and Conclusions

The objective of this study was to analyze the behaviour of TerraSAR signal as a function of sugarcane height. The radar backscattering coefficient of sampled fields was studied using ground truth measurements of sugarcane height, SPOT images, and harvest dates. The increasing trend of σ° as a function of sugarcane height is observed until a height htvd around 50 cm, corresponding to total cane height around 155 cm (depends on incidence and polarization). High correlation was observed between radar signal and NDVI index calculated from SPOT-4/5. Incidence of 47° were found to be slightly more sensitive to changes in sugarcane height at initial stages (height htvd < 50 cm). At X-band, the backscattering coefficient reaches a maximum peak value for sugarcane height htvd about 50 cm at 47°, while at 31° the peak was noted earlier (htvd = 30 cm). Cross polarization is potentially slightly better than co-polarizations for the characterization of sugarcane states. The discrimination between young and mature canes is limited to fields harvested less than 2–3 months earlier (cane heights htvd between 0 and 50 cm). X-band is not the optimal frequency to monitor crop growth on crops with significant biomass.

This study also examined the potential of different TerraSAR-X incidence angles and polarizations for mapping sugarcane harvests. Harvested fields are easily detected on SAR images if the image acquisition date is close to harvest date (ideally less than two months). Indeed, the harvest involves a decrease in the signal that can reach 7 dB (VV-37°) if the observation radar is relatively close to the harvesting date (few days). The incidences of 17° and 58° allow only partially the detection of the harvest because the decrease of radar signal after the cut is about 3 dB.

Results showed that the radar signal is very dependent on the precipitation particularly at low and medium incidence angles and for young canes. Indeed, at low and medium incidences, the soil contribution (influenced by soil moisture) to total backscattering could be important for cane heights lower than 95 cm. The soil effects are small for images acquired at high incidence angles and for sugarcanes with vegetation well developed. The decrease in radar signal for harvested fields could be reduced of 3–4 dB on images acquired after rainy period.

The very high spatial (metric) resolution of recent radar sensor (TerraSAR-X, COSMO-SkyMed and RADARSAT-2) offers great potential for mapping harvested sugarcane crop. These new SAR sensors will provide a diagnosis suited to agricultural areas where the parcels are of small size. The spatial resolution of TerraSAR images, between 1 and 3 m (for Spotlight and Stripmap modes) are well suited for sugarcane production areas dominated by small farmers as in Reunion Island with field areas of about 1 ha on average. These results appear promising for the development of simplified algorithms for monitoring sugarcane harvest regardless of meteorological conditions, which are the main limitation with optical sensors. Results obtained in this study on the potential of X-band in monitoring sugarcane growth on Reunion Island are very suitable for other environments and crops. Observations and conclusions regarding the influence of sugarcane parameters and precipitation (soil moisture) on the radar signal in X-band should be valid to other areas different from the one where these experiments have been carried out.

In the future, simulations using backscattering models (Karam, MIMICS) will be analyzed to study the dependence of radar signal at X-band to sugarcane parameters according to incidence angle and polarization. Moreover, it would be very useful to understand the contribution level of soil and vegetation layer (leaves, stems …) to the total backscattering coefficient.

## Figures and Tables

**Table 1.** Main characteristics of TerraSAR-X images used in this study.

**Incidence Angle (°)**	**Polarization**	**Imaging mode**	**Date (dd/mm/yyyy)**	**Resolution Azimuth × Range**
17	HH	Spotlight	16/03/2009; 07/04/2009; 29/04/2009; 10/05/2009	1.7 m × 1.48 − 3.49 m
	VV	Spotlight	01/06/2009; 23/06/2009	1.7 m × 1.48 − 3.49 m
	HH/VV	Spotlight	04/07/2009; 15/07/2009; 26/07/2009; 06/08/2009; 17/08/2009; 28/08/2009; 08/09/2009; 19/09/2009; 30/09/2009; 27/12/2009; 07/01/2010	3.4 m × 1.48 − 3.49 m
31	HH	Spotlight	18/03/2009; 01/05/2009; 23/05/2009	3.4 m × 1.48 − 3.49 m
	VV	Spotlight	25/06/2009	3.4 m × 1.48 − 3.49 m
	VH/VV	Stripmap	20/12/2008; 11/01/2009; 24/02/2009; 02/02/2009; 20/01/2010	6.6 m × 1.7 − 3.49 m
	HH/HV	Stripmap	22/01/2009; 13/02/2009; 18/12/2009	6.6 m × 1.7 − 3.49 m
37	VV	Stripmap	01/08/2009; 03/09/2009; 06/10/2009; 17/10/2009; 28/10/2009; 08/11/2009; 11/12/2009; 13/01/2010	3.3 m × 1.7 − 3.49 m
47	HH	Spotlight	17/05/2009	1.7 m × 1.48 − 3.49 m
	VV	Spotlight	08/06/2009; 30/06/2009	1.7 m × 1.48 − 3.49 m
	VH/VV	Stripmap	14/12/2008; 25/12/2008; 27/01/2009; 18/02/2008; 14/01/2010	6.6 m × 1.7 − 3.49 m
	HH/HV	Stripmap	16/01/2009; 07/02/2009; 12/12/2009	6.6 m × 1.7 − 3.49 m
59	HH	Spotlight	17/03/2009; 08/04/2009; 30/04/2009; 11/05/2009	1.7 m × 1.48 − 3.49 m
	VV	Spotlight	02/06/2009; 24/06/2009	1.7 m × 1.48 − 3.49 m
	HH/VV	Spotlight	05/07/2009; 27/07/2009; 09/09/2009; 01/10/2009; 28/12/2009; 08/01/2010	3.4 m × 1.48 − 3.49 m
	VH/VV	Stripmap	16/07/2009; 07/08/2009; 29/08/2009; 20/09/2009	6.6 m × 1.7 − 3.49 m

**Table 2.** SPOT-4 and SPOT-5 images used in this study.

**SAR sensor**	**Date (dd/mm/yyyy)**
SPOT-4	21/08/2008; 25/02/2009; 22/04/2009; 19/05/2009; 14/06/2009; 10/08/2009 15/08/2009; 21/09/2009; 21/10/2009
SPOT-5	24/07/2008; 21/10/2008; 17/12/2008; 21/03/2009; 24/07/2009; 22/12/2009

**Table 3.** Average values and standard deviation of sugarcane measured parameters. Htvd corresponds to the height of terminal visible dewlap.

**Day from germination**	**30**	**90**	**150**	**210**	**300**

**Cane height Htvd (cm)**	10 ± 5	66 ± 15	110 ± 20	200 ± 25	344 ± 27
**Stem number per m^2^**	5 ± 3	15 ± 8	25 ± 10	16 ± 5	10 ± 4
**Stem radius (cm)**	0.208 ± 0.04	0.586 ± 0.05	0.964 ± 0.05	1.342 ± 0.06	1.6 ± 0.06
**Stem water content (%)**	90 ± 5	90 ± 5	85 ± 4	79 ± 4	70 ± 3
**Leafs number per m^2^**	24 ± 9	72 ± 15	120 ± 20	83 ± 20	60 ± 15
**Leaf thickness (mm)**	0.26 ± 0.02	0.26 ± 0.02	0.26 ± 0.02	0.26 ± 0.02	0.26 ± 0.02
**Leaf length (m)**	0.3 ± 0.1	0.9 ± 0.15	1.4 ± 0.3	1.6 ± 0.35	1.5 ± 0.3
**Leaf width (cm)**	2.2 ± 0.25	3.7 ± 0.3	4.4 ± 0.36	4.9 ± 0.38	5.4 ± 0.4
**Leaf water content (%)**	[10–32]	[10–32]	[10–32]	[10–32]	[10–32]
